# Millennia of legal content criteria of lies and truths: wisdom or common-sense folly?

**DOI:** 10.3389/fpsyg.2023.1219995

**Published:** 2023-09-12

**Authors:** Siegfried L. Sporer, Jaume Masip

**Affiliations:** ^1^Department of Psychology and Sports Science, Justus-Liebig-University of Giessen, Giessen, Germany; ^2^Department of Social Psychology and Anthropology, Universidad de Salamanca, Salamanca, Spain

**Keywords:** history, deception detection, verbal cues, verbal content cues, content, field study, perjury cases, deception cues

## Abstract

Long before experimental psychology, religious writers, orators, and playwrights described examples of lie detection based on the verbal content of statements. Legal scholars collected evidence from individual cases and systematized them as “rules of evidence”. Some of these resemble content cues used in contemporary research, while others point to working hypotheses worth exploring. To examine their potential validity, we re-analyzed data from a quasi-experimental study of 95 perjury cases. The outcomes support the fruitfulness of this approach. Travelling back in time searching for testable ideas about content cues to truth and deception may be worthwhile.

## 1. Introduction

Susanna was a young and virtuous lady. She lived in Babylon and was married to Joacim, a rich man. Every afternoon, she used to walk in her husband’s garden. One day, during her walk, Susanna sent her maids away and asked them to shut the garden’s doors because she wanted to bath. Unbeknownst to her, she was being spied by two lecherous old men who felt lust for her. The two elders approached her and blackmailed her to have sex with them. If she refused, they would declare they witnessed her committing adultery with a young man--which would explain why she sent her maids away. She refused and screamed, and the servants of the house rushed in after hearing her screams. The two elders accused Susanna of adultery, and the next day she was sentenced to death. But she prayed to God, and God illuminated young Daniel, who decided to question each elder separately. Daniel asked the first old man under which tree he had seen Susanna meeting her lover, and the old man replied: “Under a mastic tree.” Then Daniel asked the same question to the other old man, who replied: “Under a holm oak.” Since the two men had given different answers, it became apparent to the people that their accusation against Susanna was false. Susanna was exonerated, and the two wicked elders were put to death.

This is a summary of the biblical tale of *Susanna and the elders*. We encourage the reader to read the English translation of the more detailed version in the Apocrypha *Book of Susanna* (https://t.ly/dKUZ).^1^ The story dates back to at least the 2nd century BC. We find this story fascinating because it suggests a way to detect deception in pairs (or larger groups) of witnesses or suspects–namely, asking the same unanticipated questions to each of them separately. Liars may prepare their answers to questions they can anticipate but not to unexpected questions. Therefore, if they are asked unanticipated questions, their replies are likely to be different, which will reveal their guilt. This rationale was successfully tested two millennia later by deception detection researchers (Vrij et al., 2009).

It is our tenet in this paper that century-old, or even Millenia-old writings like the tale of Susanna are good sources of inspiration for researchers seeking for deception cues. Ideas in such writings can be related to contemporary theories and transformed into formal hypotheses to be empirically tested with the methods of modern experimental psychology. Here we show how some cues examined by contemporary deception researchers resemble ideas expressed long ago by past writers, historians, and legal scholars, and we encourage researchers to scrutinize sources of these kinds in their search for promising “new” cues to deceit. We also offer examples of ideas that later turned out to be cul-de-sacs. At the end, we present a re-analysis of data of a legal doctoral thesis (Bender, 1987) that not only examined previous ideas subsequently refined by psychological researchers but also proposed to look at new aspects contemporary researchers are unlikely to be aware of.

## 2. From ancient Greece to the age of enlightenment: lie detection in scholarly and fiction writings

### 2.1. Thucydides (c. 460–400 B.C.) on the distinction between imperfect memory and partiality

Although we focus on detection of deception, in any given case errors of witnesses or suspects must also be considered as an alternative hypothesis to account for untrue statements (e.g., Sporer, 2008; Sporer and Antonelli, 2021). The distinction between error and deception has been noted throughout history. Thus, in Ancient Greece, Thucydides (c. 460–400 B.C.) already noted a “want of coincidence between accounts of the same occurrences by different eyewitnesses, arising sometimes from imperfect memory, sometimes from undue partiality for one side or the other” (Levine and Tapp, 1973, p. 1,088). Thucydides is given credit for his attempts to try to avoid these problems in describing the Peloponnesian wars, setting a standard for historiography.

### 2.2. Quintilianus (c. 35 – C. 100 AD): how to speak to be believed

In the 1st century AD, Roman orator and lawyer^2^ Marcus Fabius Quintilianus gave some advice on how to lie successfully (Quintilianus, 1991). His recommendations suggest several potential truth/deception indicators. One such indicator is (in)consistency. Quintilianus explicitly recommended orators not to contradict themselves, and stressed the need for the liar to have a good memory in order to be consistent: “the orator should bear in mind throughout his whole speech what the fiction is to which he has committed himself, since we are apt to forget our falsehoods, and there is no doubt about the proverb that a liar should have a good memory” (Quintilianus, 1991, p. 32).

*Sender inconsistencies* have been tested as a potential deception indicator in modern research. They do not seem to be strongly related to truthfulness, as results have been inconsistent across studies and are under the influence of several moderator variables (e.g., Fisher et al., 2013). Yet, Quintilianus’s advice to be consistent can still be useful to get away with one’s lies, since people *believe* lies to be less consistent than truths (Global Deception Research Team, 2006).

Some other suggestions made by Quintilianus appear to refer to *plausibility*. For instance, he wrote that “we must take care ... that our fiction is within the bounds of possibility” (Quintilianus, 1991, p. 32), and that “what we say must not be at variance with the admitted truth” (p. 32). Plausibility has been examined by contemporary deception researchers as part of the reality monitoring approach (Sporer, 2004) and more explicitly by Vrij et al. (2021). It seems to be a significant indicator of truth, though with a small effect size (*d* = 0.23; DePaulo et al., 2003), and is strongly related to *(in)consistency with the detector’s prior knowledge*, which has been shown to be one of the cues that successfully reveal deception in real-life settings (e.g., Park et al., 2002; Masip and Herrero, 2015; Levine and Daiku, 2019). The *situational familiarity* hypothesis, first put forward by Stiff et al. (1989) and more thoroughly tested by Reinhard et al. (e.g., 2011, 2012, 2013), is also explicitly based on the assessment of plausibility.

Quintilianus also wrote that falsehoods “should be connected with something that is admittedly true and should be supported by some argument that forms part of the actual case” (Quintilianus, 1991, pp. 31–32), and that the false statement should be “consistent with the persons, dates, and places involved” (p. 31). As we know both from philosophy and from research on episodic and autobiographical memory, accounts of personal events are always located in time and space, and linked to a person’s self and development (Berntsen and Rubin, 2012).

To appear believable, Quintilianus furthermore suggested to “put words in the mouth of the dead (for what they say is not liable to contradiction) or again in the mouth of someone whose interests are identical with ours (for he will not contradict)” (p. 32) and further recommended that “We must remember only to invent such things as cannot be checked by evidence” (p. 32). From contemporary research on Nahari et al.’s (2014) verifiability approach we have learned that liars may not wish to provide specific details that make an account verifiable. This type of knowledge is a two-edged sword: It helps liars to become better liars but also lie detectors to create new methods to catch them.

### 2.3. William Shakespeare’s *Hamlet*: “The lady doth protest too much, methinks” (17th century)

In William Shakespeare’s play *Hamlet* (1603), Prince Hamlet suspects that his father, the king, was murdered by Claudius, Hamlet’s uncle, who immediately married Hamlet’s mother, queen Gertrude. To verify his suspicion, Hamlet asks actors to stage a play, *The Murder of Gonzago*. The play features a death similar to the death of Hamlet’s father. Hamlet pretends to observe Claudius’s reactions to the play to determine whether he murdered his father. While Hamlet, Claudius, Gertrude, and others are watching the play, the Player Queen enthusiastically declares that if her husband dies, she will never marry again. At this point, Hamlet asks her mother: “Madam, how like you this play?,” and she replies: “The lady doth protest too much, methinks” (Shakespeare, *Hamlet*, Act III, Scene II).

Note that in Shakespeare’s times, the main meaning of “protest” was “vow” or “declare solemnly” (Macrone, 1998). To understand the role of such assertions, we must also not forget that for centuries the oath has been used to enforce truthfulness in witnesses. Hence, rephrased as a working hypothesis, Gertrude’s phrase postulates that someone who insists too strongly that she (or he) is telling the truth, or someone who makes exaggerated claims, may actually be hiding the truth. For interrogators, this hypothesis implies that they ought to explore this topic further by asking questions about different aspects of the theme (in *Hamlet*, the possibility of remarriage in case of death of the husband).

Emphasizing one’s truthfulness should make us suspicious to dig more deeply and ask related probing questions. Of course, this is only a hypothesis to be tested empirically. It does not imply that simple linguistic markers like “never” would be a valid means to differentiate lies from truth (Hauch et al., 2015). This example may also illustrate that we need to distinguish between statements by suspects and defendants in court from statements by witnesses.

### 2.4. Carlo Goldoni’s *The liar*: the ever-increasing complexity of lie construction (18th century)

In Goldoni’s (1750/1921) entertaining comedy *The liar*, similar to Ruiz de Alarcón’s (1624/2015) famous *La verdad sospechosa*, the ever-increasing complexity of lie construction is beautifully described. Lelio, always eager to please young women and to gain financial advantages, from moment to moment continuously invents new lies for his benefits to ward off questions raised by his encounters. Toward the end, the web of lies has become so complex that it can no longer be upheld and collapses.

Goldoni (1750/1921) and Ruiz de Alarcón’s (1624/2015) plays are based on the idea that liars, worried to have been caught lying, or worried about getting caught soon, may feel forced to quickly invent new lies to cover themselves. The underlying theme running throughout these plays is that (1) complex lies require continuous additions and amendments to remain credible, and (2) by doing so, liars are likely to be overburdened by the cognitive demands of this task. Thus, they will unwillingly insert contradictions into their narratives that will ultimately be revealed and lead to discovery.

Some of these notions have been considered by current deception researchers. To uphold complex lies requires “a good memory” (Quintilianus), an ancient idea that reemerges in many writings (e.g., de Montaigne, 1580/1991). Lacking a good memory, liars may engage in a “long-winded vs. issue-related reporting style” resulting in more irrelevant details and peripheral aspects (Köhnken et al., 1995). Different models of contemporary deception detection have focused on short-term/working memory components (e.g., Walczyk et al., 2014), or on the interplay of short- and long-term memory in working memory (Sporer, 2016). Other approaches have gone further and developed a host of new interview methods to elicit content cues to deception. For example, the Strategic Use of Evidence-Incremental (SUE-I) approach by Granhag et al. (2013) investigated in detail how interrogators may repeatedly probe a liar, first not revealing to interviewees the information they already have but, *peu a peu*, confronting them with this information to test for their veracity by examining whether they show contradictions.

## 3. Mittermaier’s doctrine of evidence (19th century)

Ways to assess credibility were suggested not only by ancient religious writers, orators, and playwrights, but specifically also by legal scholars and judges, who summarized their experiences from extensive casework. Their contributions demonstrate that the content-oriented approach to veracity assessment has been used throughout history. In his doctoral dissertation, Hans-Udo Bender provided a detailed review of some of these early contributions, emphasizing in particular the legal procedural writings by Mittermaier (1834), who compared the rules of evidence in several European countries at the time.^3^ The rules of evidence described principles that were supposed to help fact finders to evaluate all types of legal evidence, including eyewitness testimony (such as identification evidence), criminal allegations, and suspect statements.

Mittermaier’s recommendations were formulated in the bloomy legal language of his time, with some sentences over half a page long and difficult to understand, let alone translate. He distanced himself from prejudices toward certain religious groups, or toward women, whom he considered “capable to observe as reliably and accurately as men” (p. 345). More pertinent for us are Mittermaier’s recommendations regarding content aspects of statements. We first list a series of quotes and afterwards discuss their implications for contemporary theorizing and research using today’s terminology. Perhaps most importantly, Mittermaier (1834) recommended

“... that we believe more a person who can provide the *smallest side details of an event*, than a witness, who is unable to say anything about many circumstances, and hence demonstrates that his observation is incomplete and superficial.” (p. 347; italics added).

Mittermaier (1834) also discussed specific types of details one should expect in truthful statements, like original details, extraordinary details, or descriptions of emotions. Importantly, he also pointed out content aspects that a judge should be sensitive to because they may be indicative of a lie. For example, when a witness claims not to remember whether a victim was still alive in the morning *or* in the afternoon, we should be suspicious. But if the witness erred whether he saw him alive at 7:00 h or 7:30 h am, this may simply be due to forgetting (p. 351).

Mittermaier further addressed the question of certainty (confidence) regarding side details (pp. 351–352) and their importance for the evaluation of testimony. Certainty becomes particularly important if it concerns a “side detail that is later declared to be incorrect” (pp. 351–352). If a witness had stated this detail with “utmost certainty,” this becomes much more relevant than if the witness only “believed” the circumstance in question (p. 352).

“... side details ... must among themselves be reasonably connected to each other...,” “... in line with her/his individuality,” “and with the circumstances at the time of observation” (p. 349).

Mittermaier (1834) emphasized repeatedly that, to assess the credibility of a witness, it is also necessary to learn something about the witness’s personality (p. 294).

“The statement of a witness should be probable (*wahrscheinlich*) and ... in accordance with common laws of nature” (p. 349).

To check whether certain observations could actually have been made the way a witness described them (i.e., to formally assess “probability”), Mittermaier also recommended judges.

“... to inspect the scene of crime and/or to get help from experts” (p. 349).

Similar recommendations can also be found in later legal writings, such as in Gross’s *Handbook for investigating judges* (Gross, 1893) or in Gross’s (1898) *Criminal psychology*. For example, in his interrogations, Gross (1898) routinely “tested” witnesses’ abilities to estimate distances or time by having them gage the distance of objects (e.g., a fence) from his office window (see Sporer, 1982, and Sporer and Antonelli, 2021, for additional references).

In the next sub-sections, we examine how these propositions of Mittermaier’s (1834) *Doctrine of Evidence* relate to current theorizing and research on eyewitness testimony and deception detection.

### 3.1. Smallest side details of an event

The first quote above is probably the most important but it also entails some ambiguities that we attempt to unravel. Today we would probably replace “smallest side details” with “peripheral details.” But this focus on peripheral details leads us to potential qualifications that allow predictions from both memory research and social psychological theories on liars’ strategies.

From autobiographical and episodic memory research, we know that central details, that is, *details about the main event*, are well preserved across long time periods. For example, major actions like driving on a motorbike vs. on a bike, or the murder weapon will be correctly recalled. On the other hand, from extended conversations, people may recall only the “gist” correctly, but have often no verbatim memory and may even misremember basic contents (Neisser, 1981; Neisser and Libby, 2000). But smaller details may be forgotten faster.

But what are the smallest side details? Are they small details of the main event, for example, two, or eight or nine shots, or the buildings the person passed on the way to the scene of the crime? While a large number of shots vs. few shots should be recalled approximately correctly, buildings passed will not likely be recalled—although they could be “verifiable” details one could check. To study these distinctions, it is necessary to define main and side event and devise precise coding schemes for the analysis of verbal statements. We would recommend coding central and peripheral details separately for main and side events and investigate whether their forgetting curves differ. For likely to be forgotten details, a discrimination between memory failure and intentional distortion appears impossible. But for likely to be remembered details “I do not remember” responses or descriptions lacking details may imply deception.

On the other hand, because some people may believe that details are expected for a story to appear credible, liars may consider enriching their stories with details. However, deception researchers have argued that liars are probably reluctant to provide much detail, as this could give them away, and may not have enough cognitive resources to add such details because of the increased cognitive effort required by lying (see Granhag et al., 2015; Sporer, 2016; Vrij et al., 2016; Porter et al., 2020). These considerations are consistent with our first interpretation of Mittermaier’s perspective: truth tellers will provide more information about the main event, including important side details, than liars.

Yet, as mentioned above, smallest side details of an event can also be conceptualized as details of *side events*, such as things that happened just before or just after the core event. In an attempt to compensate for the lack of detail in the main event, liars may enrich side events with details when pressed by questions. Furthermore, because these side details may not be incriminating and hence can be reported truthfully, liars do not need to make an effort to invent them. In this way, the difficulties noted by deception researchers (Granhag et al., 2015; Vrij et al., 2016) can be overcome.

The distinction between main event and side events is also central to Sapir’s SCAN approach (for a detailed description, see Adams, 1996). According to SCAN proponents, a written narrative is supposed to be broken down into an introduction, a main part, and a conclusion, and deceptive accounts are believed to contain large proportions of text in the introduction (Driscoll, 1994). The problem is that SCAN authors disagree about the precise relative proportions of text in these sections in true and false accounts (the reader may compare Adams, 1996, with Driscoll, 1994) and provide no evidence-based guidelines on how to find the demarcation lines. Assuming that it is possible to reliably code the separation points of this tripartite structure, this would yield an interesting hypothesis. This does not imply that we encourage researchers to follow the SCAN approach (see Masip et al., 2002; Bogaard et al., 2013, for critical reviews) but only that even problematic approaches may contain elements worth considering.

Still another way to interpret Mittermaier’s statement about side details is to understand that these refer to *Superfluous Details* of the main event, which is one of the criteria in Steller and Köhnken’s (1989) Criteria-based Content Analysis (CBCA) catalog. In line with Mittermaier’s observation, Superfluous Details are considered to indicate truth by CBCA authors.

### 3.2. Other specific kinds of details and lie criteria

As already mentioned, Mittermaier (1834) discussed specific types of details one should expect in truthful statements. Contemporary researchers using the content-oriented approach also focus on specific kinds of details (like original details, extraordinary details, or descriptions of emotions). Mittermaier drew attention to content aspects that a judge should be sensitive to because they may be indicative of a lie. Such lie criteria are important because they may counteract a truth bias that may be exacerbated by using truth criteria only (Masip et al., 2009; Dukala et al., 2019).

### 3.3. Subjective certainty and spontaneous corrections

Mittermaier’s description of the role of certainty regarding side details raises two important empirical issues: (1) Is certainty regarding central vs. peripheral details related to their respective accuracies? An eyewitness study about a filmed event indicated that both accuracy and confidence covaried with the centrality of the details as well as the question format (Ibabe and Sporer, 2004). We encourage researchers to try to replicate this important finding and extend it to studies on detecting deception.

### 3.4. The witness’s personality

From a modern perspective, knowledge about the personality of the witness (or suspect) has to be considered in two different ways. First, because it can influence the way the person communicates. Thus, today, court experts on Statement Validity Assessment emphasize that the development and personality of the witness (e.g., intellectual ability, suggestibility, depressiveness, dissocial personality, histrionic personality) need to be considered to adequately assess the credibility of an individual statement (Niehaus, 2008; Volbert and Steller, 2014; Schemmell and Volbert, 2017). Examining “behavioral samples” of the witness, or using “verbal baselining” (for example, having the witness tell another similar event) have received mixed support (e.g., Dahle, 1997; Schemmell and Volbert, 2017).

Second, knowledge about the interviewees’ personality can also provide information about their habits and preferences, and thus about what behaviors are plausible. Thus, in the context of assessing suspects’ honesty, researchers have suggested that a story that does not fit with the habits and preferences of the specific suspect should raise suspicion (see Blair et al., 2012).

### 3.5. Consistency and plausibility

Mittermaier (1834) also addressed issues that today are discussed as various forms of consistency and “plausibility.”

Taken together, some of the strategies Mittermaier described have in recent years been tested in Blair et al.’s (2010, 2012) “content in context” approach, as well as in a series of studies on the situational familiarity hypothesis (Stiff et al., 1989; Reinhard et al., 2011). Both these approaches consider whether the event may have happened the way it is described considering specific contextual aspects or the evidence (e.g., knowledge about the place the event is supposed to have happened). Another approach that also compares the statement with available evidence to determine statement veracity is Hartwig et al.’s (2005) Strategic use of Evidence (SUE) technique.

We now turn to a study of court cases testing some of these ideas.

## 4. Hans-Udo Bender’s study of perjury cases (1980s)

Hans-Udo Bender’s (1987) study is a clear example of the approach we are defending here. Not only did he summarize Mittermaier’s ideas, but also other legal writings on the content of statements (e.g., Leonhardt, 1931; Peters, 1972; Bender and Nack, 1981). But H.-U. Bender also considered the writings of eyewitness psychologists Arntzen (1970/1983), Undeutsch (1967), Trankell (1972), Wegener (1981), and Köhnken (1982), who had described specific content criteria they used in their daily work as experts on credibility assessments. H.-U. Bender attempted his own integration of these approaches into a set of credibility criteria which he tested in a validation study of courtroom cases of perjury. The goal of the study was to find out whether a content analysis of the information available to judges in the form of witness statements would discriminate true statements from perjuries.

As shown in Table 1, H.-U. Bender distinguished singular reality criteria (e.g., Extraordinary Details), global reality criteria (Homogeneity and Structural Equality), and criteria for repeated statements (Supplementation and Constancy). While some criteria (e.g., Detailedness, Descriptions of Emotions) resemble some of the CBCA items later encountered in Steller and Köhnken’s (1989) integrative summary, others are less likely to be known to English speaking readers. Most of the criteria mentioned are considered reality indicators.^4^

**Table 1:** Proportions of reality criteria and fantasy signals, odds ratios, effect sizes g, 95% CIs, diagnosticity, base rates, and prevalences.

Criteria	*p*_Truth_	*p*_Lie_	DOE	*g*	CI95_Lo_	CI95_Hi_	*p*(ChiSQ)	Diagnosticity	Prevalence in truths	Prevalence in lies
*Singular reality criteria*										
Detailedness (of main/side event)	0.804	0.158	1	1.65	1.20	2.11	<0.001	5.09	0.80	#
Extraordinary details	0.150	0.025	1	0.44	0.03	0.85	0.036	6.00	0.15	#
Description of emotions	0.265	0.155	1	0.23	−0.17	0.64	0.255	1.71	0.27	#
Complications	0.170	0.050	1	0.38	−0.03	0.79	0.065	3.40	0.17	#
Misunderstood details	0.000	0.000	0	X	X	X	ns.	X	0.00	#
Anchoring (in concrete life situations)	0.115	0.050	1	0.23	−0.17	0.64	0.253	2.30	0.12	#
Voluntary description of unflattering details/self-accusations	0.075	0.000	1	0.38	−0.03	0.79	0.069	> 4	0.08	#
*Unweighted Mean*	*0.226*	*0.063*	*1*	*0.55*	*0.14*	*0.96*	*X*	*X*	*0.23*	*#*
*Global reality criteria*										
Homogeneity: Internal logical consistency and laws of nature; not case facts	0.735	0.190	1	1.28	0.85	1.71	<0.001	3.87	0.74	#
Structural equality regarding case relevant details	0.470	0.050	1	1.05	0.62	1.47	<0.001	9.40	0.47	#
*Criteria for repeated statements*										
Supplementation	0.395	0.145	1	0.58	0.16	0.99	0.007	2.72	0.40	#
Constancy	0.510	0.240	1	0.57	0.15	0.99	0.007	2.13	0.51	#
*Fantasy signals (lie criteria)*										
Abstractness (details at best in side events)	0.157	0.763	−1	−1.52	−1.90	−1.15	<0.001	4.87	#	0.76
Exaggeration of own truthfulness	0.020	0.215	−1	−0.66	−1.05	−0.28	0.002	10.75	#	0.22
Inhomogeneity	0.150	0.570	−1	−0.98	−1.35	−0.60	<0.001	3.80	#	0.57
Disrupted structure	0.020	0.380	−1	−1.05	−1.43	−0.68	<0.001	19.00	#	0.38
Freudian slip	0.000	0.070	−1	−0.41	−0.80	−0.02	0.048	> 3	#	0.07
*Fantasy signals for repeated statements*										
Emaciation (reduction of main event details over time)	0.055	0.355	−1	−0.82	−1.20	−0.44	<0.001	6.45	#	0.36
Inconstancy/Stereotypicality	0.115	0.380	−1	−0.66	−1.04	−0.27	0.002	3.30	#	0.38

For all criteria but Detailedness and Abstractness, n_Truth_ = 53 and n_Lie_ = 42, with a base rate of true accounts of 53/(53 + 42) = 0.56; for Detailedness and Abstractness, n_Truth_ = 51 and n_Lie_ = 38, with a base rate of true accounts of 51/(51 + 38) = 0.57.

*p*_Truth_: proportion of truthful accounts with the criterion;  *p*_Lie_: proportion of deceptive accounts with the criterion; DOE: observed direction of the effect, with 1 indicating stronger presence of the criterion in truthful accounts and − 1 indicating stronger presence of the criterion in deceptive accounts; g: unbiased Hedge’s g; CI95_Lo_ and CI95_Hi_: lower and higher bounds of the 95% confidence interval for *g*; *p*(ChiSQ): *p* value for the Chi square; Diagnosticity = *p*_Truth_/*p*_Lie_ for truth criteria and *p*_Lie_/*p*_Truth_ for fantasy signals; X: impossible to calculate; #: not relevant.

Importantly, H.-U. Bender also included “fantasy signals” whose presence were to be interpreted as lie criteria (see Bender and Nack, 1981, for detailed descriptions and examples from trial interrogations and strategies used by judges to test for the presence of individual criteria). Fantasy signals were either defined as antinomies of some of the truth criteria mentioned (Abstractness, Inhomogeneity, Disrupted Structure, Emaciation [*Abmagerung*], Inconstancy/Stereotypicality) or taken from Bender and Nack (1981), *viz.* Exaggeration of Truthfulness and Freudian Slips.

H.-U. Bender also reported prevalence rates of reality and lie criteria in true and false statements. Prevalence rates can guide the user of these criteria to gauge their practical utility.^5^ If a reality criterion is only encountered rarely in the universe of cases, its practical utility will be undermined even if it has a large odds ratio or a large Hedges’s *g*.

This emphasizes the point that a specific criterion may well be diagnostic of truth status in a given case, but may nonetheless not be very useful in the majority of cases if it is only rarely encountered in this type of case. Furthermore, Bender suggested an index for the evidentiary value of his criteria (*Indizstärke*) in the form of a diagnosticity ratio. For reality criteria, diagnosticity equals the percentage of true accounts with the criterion present divided by the percentage of lies with the criterion present. For fantasy signals (lie criteria), diagnosticity equals the percentage of lies with the criterion present divided by the percentage of true accounts with the criterion present.

A unique aspect of H.-U. Bender’s study is that it also explored the discriminative value of co-presence/co-absence of a small set of criteria (criteria combinations).

### 4.1. Bender’s method

To test the validity of his criteria, H.-U. Bender conducted an archival analysis of courtroom cases of intentional perjury that were selected to avoid issues of circular validation often present in sexual abuse allegations.

H.-U. Bender’s materials were from trials in which one or several witnesses had testified to help a defendant—a relative, friend, or work colleague—not to be convicted. When judges suspected intentional perjury had occurred, they charged these witnesses. This resulted in 95 purported perjury cases for analysis, with 53 witness statements judged to be truthful (no perjury) and 42 statements leading to convictions of perjury. More than half of the cases were validated by objective evidence (like a log book of car travel times), or in many occasions by later confessions of perjury. In a few cases, contradictory statements by other witnesses and other suspicious aspects of the case also led to a conviction of perjury by the trial judges.

To demonstrate at least some effort to establish inter-coder reliability, 12 statements were each coded by three experienced practitioners and by H.-U. Bender himself. Singular reality criteria in transcripts of statements were coded by marking relevant passages as “clearly present.” Bipolar criteria (e.g., Detailedness vs. Abstractness, Homogeneity vs. Inhomogeneity) were coded separately as truth and lie criteria, respectively.

Although no formal statistical values for inter-coder reliability were calculated, percentages of agreement were highly satisfactory for these cases (see the Appendix in Bender, 1987, pp. 190–213). However, the main problem is that the full set of cases were apparently coded only by the author, who might not have been blind to the ultimate case outcomes (although presumably the outcome was in other parts of the case files than the statements). Therefore, the reader should bear this caveat in mind in considering the following results.

### 4.2. Results: reanalysis of Bender’s data

We reanalyzed Bender’s (1987) data to more precisely assess the potential of the verbal content criteria that he collected and (re-)defined. Table 1 displays the proportions of criteria rated present in the 53 true and the 42 perjury cases (except for Detailedness and Abstractness, for which the number of cases was 51 and 38, respectively), with a base rate of true accounts of 0.56 (0.57 for Detailedness and Abstractness). We also calculated the odds ratios, the log(OR), the bias-corrected standardized mean difference *g*, the diagnosticity of each criterion and the prevalence rates of the criteria for truths and lies.

Among the singular criteria, only Detailedness and Extra-ordinary Details discriminated significantly between truths and lies.^6^ Prevalence was high for Detailedness but not for Extra-ordinary Details. Although several other singular criteria had medium to large effect sizes in the expected direction, they were not significant. Hence, relying on specific singular criteria appears dangerous.

Global reality criteria and criteria for repeated statements were all significant and had large or medium effect sizes in the predicted direction (all *p*s < 0.01), with prevalence rates between 0.40 and 0.74. All fantasy signals were significant, with medium to large effect sizes, but some had low prevalence rates (in particular Exaggeration of own truthfulness and Freudian Slips, see Table 1). Therefore, they do not have much practical utility.^7^ Fantasy signals for repeated statements were highly significant with large effect sizes but had somewhat low prevalence rates. Diagnosticity ratios were reasonably high for most criteria, in line with the effect sizes reported (Table 1).

It is impossible to summarize the complexity of H.-U. Bender’s analyses regarding the co-occurrences of two or three criteria in true and in false statements. Also, not enough statistical details were provided for a full re-analysis of the data, nor was the sample large enough to analyze for higher-order dependencies. Nonetheless, the best example of the potential fruitfulness of this approach is the demonstrated co-occurrence of Detailedness and Homogeneity. This combination was observed in 62.5% of true and only 2.5% of false statements (*p* < 0.001; *g* = 1.57). Conversely, Abstractness and Inhomogeneity were found in 2.0% of true and 45.0% of false statements, respectively (*p* < 0.001; *g* = −1.23). Both combinations had diagnosticities >20.0 and good practical utilities (prevalence = 0.63 and 0.45, respectively). The criteria combinations investigated by H.-U. Bender also showed significant discrimination (all *p*s < 0.02) and high diagnosticity values but differed in practical utility. However, these findings may be specific to the perjury cases studied and should not be overinterpreted.

### 4.3. Discussion

#### 4.3.1. Discussion of our reanalysis of Bender’s data: “Lie signals,” criteria combinations, and diagnosticity

A potential danger of finding many criteria in an account is that this may induce a truth bias in the ultimate evaluation of a case (Niehaus, 2001; Dukala et al., 2019), because all CBCA criteria are considered truth criteria (see Masip et al., 2009). Bender (1987), like several other scholars (Mittermaier, 1834; Hellwig, 1951; Bender and Nack, 1981, 1995), proposed specific “lie signals” judges should pay attention to and investigate further. Among these signals were both nonverbal cues but also *lie criteria*, that is, content qualities that are predicted to be found more often in invented than in self-experienced accounts. We encourage future researchers to further explore the criteria mentioned by these and other authors as well as to search for and test new lie criteria (e.g., Köhnken et al., 1995; Niehaus, 2001; Nahari et al., 2019). Ancient texts written by religious writers, historians, orators, novelists, playwrights, and legal scholars are a good source of ideas that can be reformulated as testable working hypotheses.

In his archival analysis of perjury cases, Bender (1987) proposed that specific combinations of criteria might indicate truthfulness. In his explorative analyses, both two- and three-way combinations were found to discriminate. In statistical terms, the notion of criteria co-occurrence implies that multiway frequency analyses for criteria coded in binary form (presence = 1 vs. absence = 0) could be conducted to test whether the co-presence of two criteria (criterion X: 1 and criterion Y: 1) is more indicative of truth than other combinations (1/0; 0/1; 0/0). Combinations suggesting truthfulness may involve not only the co-presence of criteria; they can also consist of the presence of a certain criterion and the *absence* of another one.

Hommers (1997) and Hommers and Hennenlotter (2006) refined H.-U. Bender’s ideas and applied them to larger experimental data sets. Specifically, they re-analyzed accounts collected by Steller et al. (1992) and cross-validated data of Wolf and Steller (1997) considering the co-occurrence of criteria. The details of these analyses are beyond the scope of this paper, but we encourage researchers to further explore similar approaches. Note that this perspective has been used for decades in medical diagnoses, where specific symptom combinations allow for a much better diagnosis than the occurrence of individual symptoms.

For rating scales, a multiplicative combination of pairs of criteria might be used to predict truthfulness. However, such combinations should be based on theory, not just used in an ex-post-facto exploratory fashion.^8^ We encourage authors to inspect the intercorrelation matrices of their previous research on any kind of content cues to cross-validate (or disprove) our arguments (and to send us the results).

Bender (1987) also emphasized that the practical relevance or diagnosticity of a criterion (or of a specific combination of criteria) depends on its prevalence rate. However, although a specific criterion may not have a large effect size due to a floor effect in a given domain, it may nonetheless be very important in the analysis of a particular case. For example, *Details Misunderstood* may only occur in individuals with a poor understanding of the event, and even there very infrequently, even if the account is truthful. But if *Details Misunderstood* do occur, they may be highly indicative of the truthfulness of the statement analyzed.

#### 4.3.2. The many facets of (In)consistency

Goldoni’s (1750/1921) play summarized above, which highlights the risk for liars to contradict themselves, is just one example to show that the basics of today’s sophisticated approaches are part of our cultural heritage. These ideas have to be developed further and differentiated. Here we just note the many aspects of “*consistency*” that have been “rediscovered” again and again in different areas and approaches. Different authors have addressed some of these aspects more thoroughly than others depending on their area of specialty, for example, in memory or in deception research. Ultimately, what would be needed is a taxonomy of the many facets of “consistency” (or inconsistency).

Consistency between reports is not only an issue in deception detection but in any type of witness or suspect statement. Although people usually use the term “consistency,” the examples given in cases often concern *in*consistency. While this point may be trivial, it has implications for coding a narrative account. Consistency concerns the whole account and hence allows only a binary coding (0 vs. 1). Inconsistencies may refer to specific parts and aspects and hence allow more differentiated, and hence more sophisticated coding of reliability (Hauch et al., 2015). Inconsistencies can be coded via frequencies or via ratings (e.g., 1 to 7; 0 to 4), which may result in higher statistical power compared to a mere dichotomy implied by the term “consistency.”

Here we only list a series of different aspects of (in)consistency:

- error vs. deception,
- consistency with laws of nature,
- consistency between people,
- internal (logical) consistency within one single statement/interview,
- consistency across different statements of the same person,
- consistency of statements across different members of a group (who may or may not have communicated with each other), and
- consistency regarding witnesses vs. suspects.

We note that within the last two decades there has been a shift in emphasis from studying deceptive statements of witnesses to finding new methods of interviewing/interrogation of suspects. While government agencies have always financed research on the latter, the events of September 11th, 2001, and the subsequent U.S. Government reactions, appear to have instigated funding for new methods to deception detection (including detection of intent). In the United States, the U.S. High-value Detainee Interrogation Group (HIG) research program is an example (see Meissner et al., 2017; Brandon et al., 2019), but there have also been cross-national collaboration programs. From a free (and open) science perspective, a potential problem is that some of this research may be classified (though this is not the case for the HIG program) and thus not subject to peer review and critique (for example, research on deception at airports, at immigration offices, but also deception by asylum seekers or insurance claimants). Governments may fall prey to consultants who have an interest to make their methods not publicly known, which may also lead to large sums of money spent on pseudoscience (Denault et al., 2020).

Any of the (in)consistencies noted above may occur in combination with any of the others, giving rise to complex higher order interactions (conceptually and statistically). Results may differ across such subgroups of studies and hence comparing studies across groups may not be meaningful. This may be particularly true for content-oriented approaches.

A final point we want to address is:

- consistency/inconsistency with known case facts or evidence.

While studies address questions about these consistencies, we need to ask ourselves who are legally entitled to address these issues in real cases. In a democracy, these decisions are reserved to the judiciary, that is judges, magistrates, or juries. To what extent are forensic experts, or (poorly trained) police officers, allowed to have access to case facts and incorporate them in their decision? In some legal systems, experts are often denied permission to testify because their testimony may invade the province of the jury. Hence, to remain impartial, they should not have access to other case facts than the specific issue to be evaluated. For example, for expert evaluations of forensic evidence like fingerprints, Dror et al. (2006) argued that experts should only be sent sets of fingerprints to match but no other case information that could lead to a series of contextual biases (for an integrative model, see Dror, 2020). Bogaard et al. (2013) have demonstrated that such biases may also operate in content approaches to detect deception. Thus, contextual information can help discover the truth but may also bias assessments at all stages of the judicial process, from police investigations to judge or jury decisions.

## 5. Conclusion

We encourage contemporary researchers to look back in history and consider writings from different disciplines from our cultural heritage, in particular legal writings, to search for solutions to the problem of deception detection. Throughout history, error and deception were always considered as threats to the validity of the content of statements. Our review of such sources is only cursory and eclectic, but we nonetheless offer the following conclusions:

(1) Some of these writings offer interesting suggestions regarding cues to deception and interrogation strategies to discover them. These suggestions can be related to contemporary theories of deception detection and reformulated into testable research hypotheses, including potential moderator variables sometimes not considered in contemporary research.
(2) Our cursory review demonstrates that many ideas in the current literature may not be as original as it may first seem, and hence we recommend that terms like “We are the first to investigate...” should simply be avoided.
(3) While some of the cues described, in particular nonverbal ones, are often contradicted by empirical research, the content approach appears to have been the primary successful approach throughout history, not a 20th century or last decade invention.
(4) Conclusions like “The statement contained more details” are too unspecific and need to be qualified by specific types of details that may be valid only in subareas of deception detection.
(5) Noteworthy, certain types of details may only be found in descriptions of the main event, others in side events. The challenge will be to find precise definitions of main and side events.
(6) Nonetheless, some limitations have to be noted. Looking around the globe ignores cultural, language-specific and country-specific legal differences that we have not addressed.
(7) Some of the content cues mentioned may be idiosyncratic to certain types of legal contexts that may not apply to others: statements of witnesses vs. suspects, videotaped vs. oral vs. written statements, criminal vs. civil vs. other types of legal cases (family court hearings, harassment and abuse allegations, insurance fraud, disability claims, medical symptom reports, asylum applications, etc.).

Finally, comparing writings from experts in certain fields with knowledge from experimental psychological laboratory research is not novel (*cf.* King and Dunn, 2010, and the critical analysis by Blair et al., 2012). Blair et al. specifically address correspondence and coherence aspects within a statement and statements of suspected others and relate their arguments to philosophical writings on correspondence theory of proof (see Dunwoody, 2009). These aspects are beyond the scope of this paper. We have pointed out that there are many forms of consistency that should be further explored, and emphasize that the context of a statement needs to be considered (Masip and Sánchez, 2019; Sánchez et al., 2021), as exemplified by Reinhard et al.’s (2011) admonition “Listening, not Watching.” On the other hand, we have also pointed to the biasing effects of contextual information that may affect forensic decision makers that are difficult to avoid (Dror et al., 2006; Dror, 2020). Perhaps, research institutes that are independent from governments and funding pressures could best fulfill this call for objectivity.

## Author contributions

SLS conceived this paper and re-analyzed Bender’s (1987) data. SLS and JM independently searched for historical sources and equally contributed to writing the manuscript. All authors contributed to the article and approved the submitted version.

## Funding

Article processing charges are covered by Universidad de Salamanca, *Programa V: Difusión de Resultados de Investigación*.

## Conflict of interest

The authors declare that the research was conducted in the absence of any commercial or financial relationships that could be construed as a potential conflict of interest.

## Publisher’s note

All claims expressed in this article are solely those of the authors and do not necessarily represent those of their affiliated organizations, or those of the publisher, the editors and the reviewers. Any product that may be evaluated in this article, or claim that may be made by its manufacturer, is not guaranteed or endorsed by the publisher.

## Footnotes

^1^This tale was originally placed at the beginning of the *Book of Daniel*, but it was later removed by the compilers of the Hebrew Bible, thus becoming part of the Apocrypha. Consequently, modern Jewish and Protestant Bibles do not have this story. Very old Greek texts contain it before Daniel 1:1 as a prolog, and it is found as Chapter 13 in the *Book of Daniel* in the *Vulgate*, as well as in modern Catholic and Orthodox Bibles [https://simple.wikipedia.org/wiki/Susanna_(Book_of_Daniel)]. For a history of the reception of the story and bibliographical details, see Tkacz (2008).

^2^Born in the Roman province of Hispania Tarraconensis (currently in the Rioja territory of Spain) Quintilianus was educated in Rome to study rhetorics, that is, the art and techniques of convincing people in oral speech. Quintilianus applied this knowledge practicing law before the courts, but his main focus was on the personal education of speakers (Clarke, n.d.). Late in his life he wrote a 12-volume work on rhetorics (around 95 AD).

^3^In Germany, the rules of evidence were replaced by the free evaluation of evidence. In other countries some rules still exist (like Frye, Daubert, and the *Federal Rules of Evidence* in the U.S.).

^4^H.-U. Bender preferred the term “subjective truth” because statements could also contain unintentional errors.

^5^H.-U. Bender referred to this as “*Praxisrelevanz*.”

^6^In H.-U. Bender’s dissertation, the Chi-Square tests were not reported. We recalculated all significance tests using Chi-Square tests and the associated exact *p* values, which slightly differ from those reported in the dissertation. We calculated effect sizes from our Chi-Square values as described in Lipsey and Wilson (2001).

^7^Exaggeration as a fantasy signal, which resembles the *Hamlet* example described above, may be more likely to appear in perjury trials than in other cases. Since the truthfulness of a witness’s statement is at the very center of attention, defendants may emphasize their truthfulness to counteract a judge’s suspicion. Regarding Freudian Slips, we have an unpublished pilot study in our files in which the coder gave up searching for them after 50 out of 184 cases. The coder had encountered a single instance—but in a true account. For further references on Freudian slips, see Sporer (2016).

^8^For example, if all pairwise combinations of the 19 CBCA criteria were to be used, 19*19 = 361 criteria combinations would have to be added to the 19 original criteria in the prediction equation. Of course, an extremely large number of cases would be necessary to test and cross-validate such a model, and even larger data sets would be necessary to explore higher-order co-occurrences.
